# Improving stroke outcomes through progressive implementation of stroke unit care in Brazil: a longitudinal observational study

**DOI:** 10.3389/fstro.2026.1825941

**Published:** 2026-05-25

**Authors:** Giovani Noll, Artur Francisco Schumacher Schuh, Lenise Valler, Andrea Garcia de Almeida, Rosane Brondani, Magda Carla Ouriques Martins, Gustavo Weiss, Angélica Dal Pizzol, Letícia Costa Rebello, Luiz Antônio Nasi, Sheila Cristina Ouriques Martins

**Affiliations:** 1 Neurology Department, Hospital de Clínicas de Porto Alegre, Porto Alegre, Brazil; 2 Universidade Federal do Rio Grande do Sul, Porto Alegre, Brazil; 3 Neurology and Neurosurgery Department, Hospital Moinhos de Vento, Porto Alegre, Brazil; 4 Neurology Department, Hospital de Base do Distrito Federal, Brasília, Brazil; 5 Emergency Department, Hospital de Clínicas de Porto Alegre, Porto Alegre, Brazil; 6 Cardiology Department, Hospital Moinhos de Vento, Porto Alegre, Brazil

**Keywords:** delivery of health care, developing countries, low- and middle-income countries (LMIC), public health, stroke, stroke unit care

## Abstract

**Introduction:**

Stroke remains a leading cause of death and disability worldwide, with a disproportionate burden in low- and middle-income countries (LMICs). Although stroke unit (SU) care improves outcomes, evidence from LMIC settings is still limited. We evaluated the impact of three sequential stroke-care models implemented in a Brazilian public university hospital.

**Patients and methods:**

This longitudinal observational study included 1,889 patients with ischemic or hemorrhagic stroke across three care models: before stroke unit (BSU), acute stroke unit (ASU), and comprehensive stroke unit (CSU). Demographic characteristics, stroke subtype, baseline severity, imaging metrics, and outcomes were collected using retrospective and prospective approaches. Primary outcomes were 90-day case fatality and functional status assessed by the modified Rankin scale (mRS). Secondary outcomes included door-to-CT time, thrombolysis rates, pneumonia, access to rehabilitation, and length of stay. Multivariable logistic regression was used to identify predictors of mortality and excellent functional outcome (mRS 0–1).

**Results:**

The implementation of structured stroke-care models was associated with significant improvements in outcomes. Functional independence at 90 days (mRS 0–2) increased from 45.6% (BSU) to 60.3% (ASU) and 56.3% (CSU) (*p* < 0.001), while case fatality declined from 24.3 to 10.2% and 7.7%, respectively (*p* < 0.001). Key quality indicators improved substantially: mean door-to-CT time decreased from 527 to 170 and 107 min (*p* < 0.001), thrombolysis rates increased from 0 to 14.1% and 22.2% (*p* < 0.001), and post-stroke pneumonia rates declined from 29.4 to 16.3% and 12.5% (*p* < 0.001). In multivariable analyses, older age and higher baseline NIHSS were independently associated with increased mortality, whereas intravenous thrombolysis and SU care were associated with reduced odds of death. SU care was associated with more than a threefold increase in the likelihood of excellent functional outcome, while thrombolysis remained the strongest predictor (OR 6.19).

**Conclusion:**

Stepwise implementation of structured stroke-care models significantly reduced mortality and improved functional outcomes in this LMIC setting. These findings support the effectiveness and scalability of SU—particularly comprehensive models—as key strategies to strengthen stroke systems of care.

## Introduction

Stroke stands as a major cause of both disability and mortality worldwide, with low- and middle-income countries (LMICs) carrying the greatest burden despite limited healthcare infrastructure (GBD 2019 Stroke Collaborators, 2021; Owolabi et al., 2021). Prevention alone cannot address this inequity and evidence-based interventions should be implemented for the entire population, providing a pressing need for a substantial modification in public health strategies and stakeholders planning (Saini et al., 2021).

Stroke units (SUs)—dedicated hospital areas staffed by trained multidisciplinary teams and delivering a standardized bundle of care—are central to organizing effective stroke management. Their implementation is feasible in LMIC (Langhorne et al., 2012) yet most supporting evidence originates from high-income countries. In resource-limited settings, barriers such as restricted access to hospital care, workforce shortages, and infrastructural constraints remain critical. Notably, an international survey found that 91% of hospitals in high-income countries had a SU, compared with only 18% in low-income countries (Owolabi et al., 2021).

The Hospital de Clínicas de Porto Alegre (HCPA), a public university hospital in Southern Brazil, implemented intravenous thrombolysis in December 2005 (Nasi et al., 2014; Martins et al., 2013) with hospital resources and has been working with emergency stroke care protocols since then. In October 2012, it was one of the first hospitals licensed as a Stroke Center in Brazil by the Ministry of Health. Since then, HCPA has functioned as a comprehensive stroke unit (CSU) with integrated early rehabilitation. This study evaluates clinical outcomes and quality-of-care indicators across three successive models of stroke care implemented step by step at HCPA.

## Methods

### Study design, exposures, and the line of stroke care

**Figure 1:**
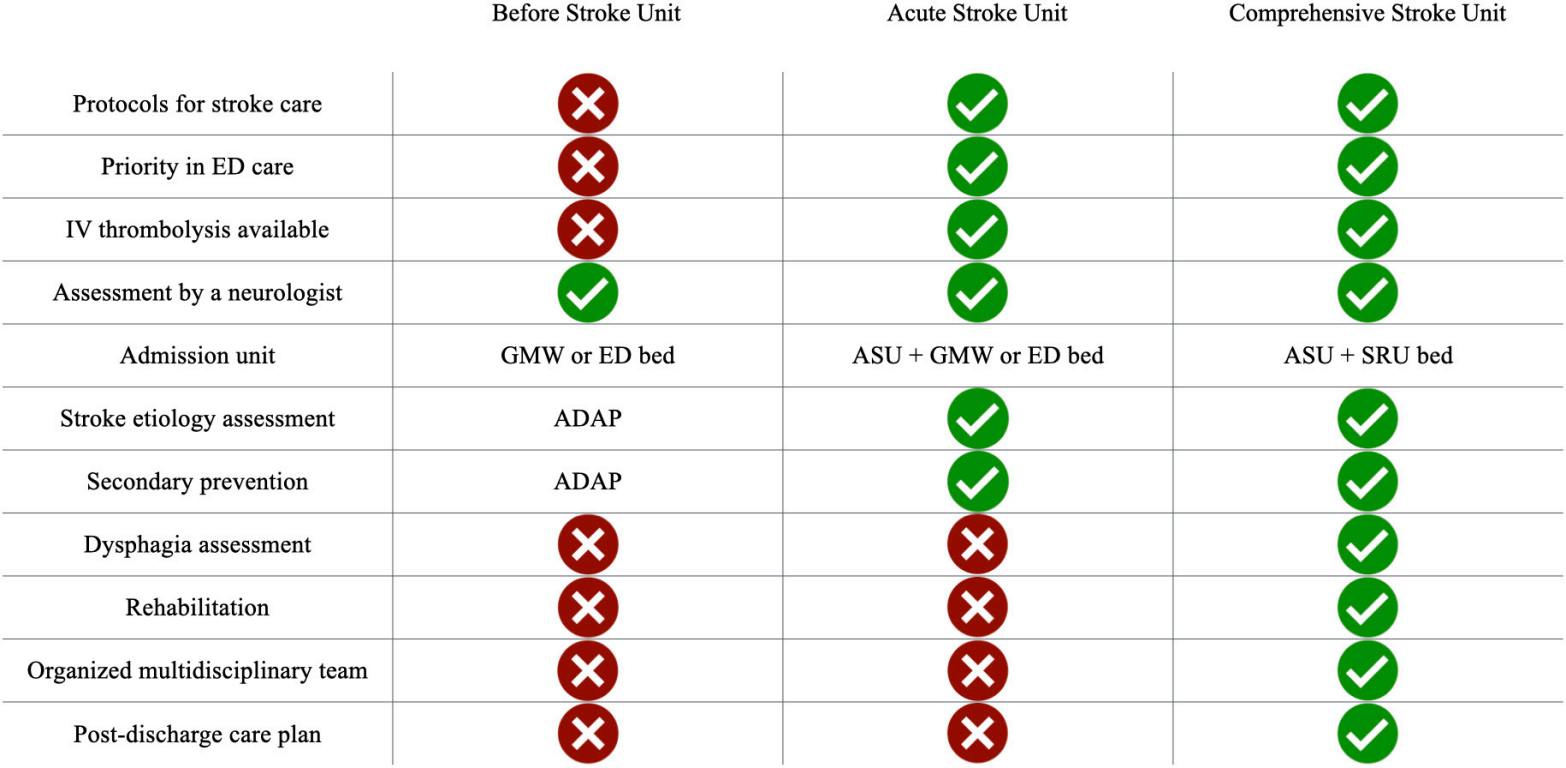
Main characteristics and differences among the strategies of care. ADAP, at the discretion of the attending physician; ASU, acute stroke unit; ED, emergency department; GMW, general medical ward; IV, intravenous; SRU, stroke rehabilitation unit.

We conducted a longitudinal observational study with retrospective and prospective components, comparing three stroke-care strategies at HCPA. Admissions and outcomes were analyzed across three periods, each defined by a distinct model of care (Figure 1):

- Before stroke unit (BSU): data was retrospectively collected from medical records of patients admitted in 2005. This phase preceded organized stroke care. There was no prehospital protocol, and stroke lacked priority in the emergency department (ED). Patients arrived independently or via emergency services but were triaged without urgency. Computed tomography (CT) imaging was delayed, no reperfusion therapy was available, and admitted patients were managed in general wards or ED beds. Etiologic workup and secondary prevention were variable, screening for dysphagia (Azer et al., 2026) was absent, and patients rarely received rehabilitation or referral for post-discharge therapy.
- Acute stroke unit (ASU): data collected prospectively from patients admitted between 2009 and 2012. A specialized ED-based Vascular Unit, launched in December 2005 (Nasi et al., 2014; Martins et al., 2013), provided hyperacute management for vascular emergencies under a trained team, including 24/7 on-call stroke neurologists. Hyperacute ischemic stroke patients became eligible for thrombolysis. Patients with acute intracranial hemorrhage and subarachnoid hemorrhage were also admitted to this unit at first. After initial management and monitoring (48 to 72 h), patients were transferred to general ward beds, where they received care from a stroke neurologist, albeit without an organized multidisciplinary team. Speech therapy, swallowing assessments, and motor physiotherapy were rarely available. The ASU established HCPA as a regional thrombolysis hub, prompting reorganization of prehospital triage. Consequently, local and regional prehospital services underwent reorganization to prioritize assistance for patients with suspected stroke, particularly those eligible for thrombolysis. During this period, approximately 40% of stroke patients admitted in Porto Alegre hailed from nearby cities by referrals from smaller hospitals. Patients within the 4.5-h window from symptom onset were predominantly transported to HCPA, while those with neuroimaging evidence of hemorrhagic stroke were primarily directed to other city hospitals, leading to a shift in the distribution of stroke subtypes admitted to HCPA in comparison to the BSU period.
- Comprehensive stroke unit (CSU): data was collected prospectively from patients admitted between 2013 and 2015. This period started following the launch of the National Stroke Policy by the Ministry of Health and the licensing of the HCPA as a Stroke Center. During this period, patients initially received care at the ASU, including thrombolysis when indicated, and subsequently were transferred to an inpatient rehabilitation unit with an organized multidisciplinary team comprising stroke neurologists, experienced nursing staff, a speech therapist, a physiotherapist (available 7 days a week, during the day shift), a nutritionist, a social worker, and additional support from a pharmacist and psychologist as needed. Dysphagia screening became routine practice, conducted by a speech therapist using the Gugging Swallowing Screen (GUSS) (Trapl et al., 2007). Specific rehabilitation needs were assessed at discharge, and individualized post-discharge care plans were formulated for each patient. During this period, all suspected ischemic strokes within 12 h were preferentially transported to HCPA, while confirmed hemorrhagic strokes continued to be referred mainly to other city hospitals.

### Study setting

**Figure 2:**
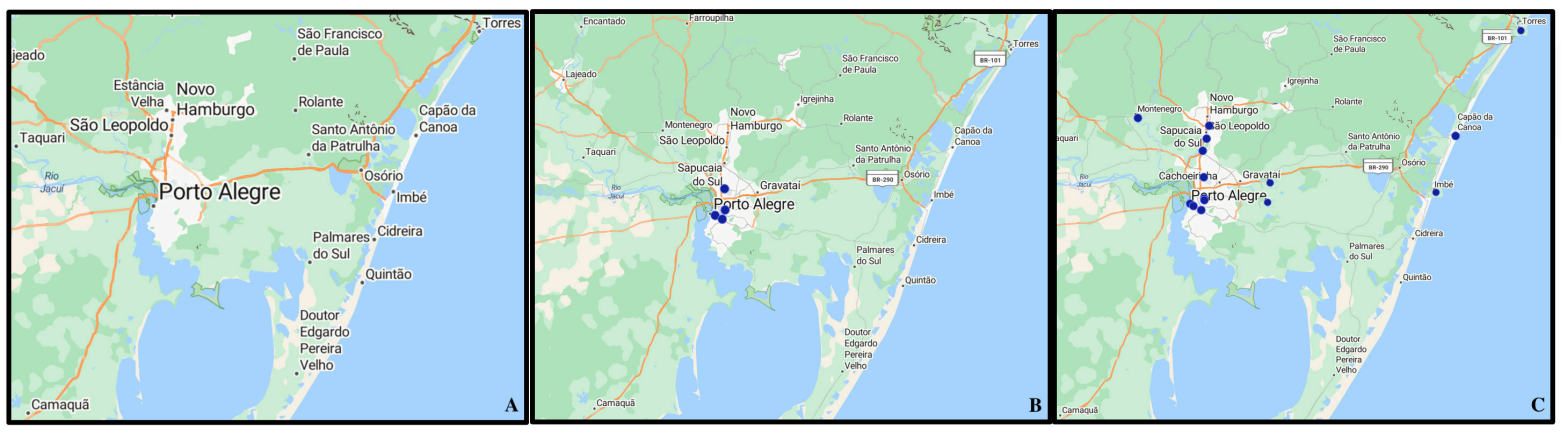
Acute stroke network in the region. Blue dots in the map represent stroke centers available in each period. **(A)** Before stroke unit (BSU—Baseline Scenario): twenty-six hospitals with CT capability managed all stroke patients, who were treated in general emergency departments and admitted to general wards. There were no dedicated stroke centers in the region. **(B)** Acute stroke unit (ASU—Pilot Phase): four hospitals, designated as acute stroke centers and participating in a Ministry of Health pilot program, received all stroke patients in the region. **(C)** Comprehensive stroke unit (CSU—post-policy implementation): following the implementation of the national stroke policy, 16 stroke centers with dedicated stroke units (acute + rehabilitation) were established, enabling a regionalized distribution of stroke patients across the network.

The HCPA, a tertiary public university hospital, boasted 850 beds during the study period (currently expanded to 1,000 beds). Serving as a regional hub for acute stroke care within the public health system, the hospital admitted 248 stroke patients in 2005, and this figure varied in subsequent years based on the configuration of the acute stroke network in the region (Figure 2). Today it functions as a high-volume Stroke Center admitting around 1,000 stroke patients per year, with an ED-based ASU and a dedicated inpatient rehabilitation unit staffed by a comprehensive multidisciplinary team.

Following implementation of Brazil's National Stroke Policy in 2012, the number of public stroke centers in this 4-million-inhabitant region increased from 4 to 16, improving system capacity. Thrombolysis volumes at HCPA have since stabilized at 65–80 cases/year. The stroke team includes four stroke neurologists supervising onsite neurology residents, providing a 24/7 coverage supported by telemedicine when needed.

HCPA has maintained a specialized outpatient stroke clinic since 1998, with 80% of patients attending at least one post-discharge visit. Patients with lacunar stroke, transient ischemic attack, or minor stroke who complete etiologic evaluation in-hospital are referred to primary care. Access to outpatient rehabilitation remains limited due to public-system constraints and logistical barriers.

### Data collection, sample, variables, and outcomes

All patients diagnosed with stroke or transient ischemic attack at HCPA were included. Diagnosis was confirmed by a certified neurologist using clinical and neuroimaging criteria (Easton et al., 2009; Sacco et al., 2013). Stroke mimics, defined as non-vascular conditions that present with acute focal neurological deficits, simulating an acute ischemic stroke but lacking evidence of cerebral infarction on neuroimaging (MRI/CT), in which further investigation did not meet the above criteria and/or in which a clear alternative etiology was identified (H Buck et al., 2021), were excluded.

Extracted variables included demographics (age, sex, race), stroke subtype [ischemic (Sacco et al., 2013) or hemorrhagic (Connolly et al., 2012; Sacco et al., 2013)], comorbidities, baseline National Institutes of Health Stroke Scale (NIHSS), arrival mode (by ambulance vs. others), and the received bundle of care (BSU, ASU, CSU). Primary outcomes were case fatality and 90-day modified Rankin scale (mRS). Additional quality indicators included door-to-CT time, alteplase administration among ischemic stroke patients, in-hospital pneumonia rate [defined based on a combination of clinical, radiological, and laboratory parameters (Smith et al., 2015)], dysphagia screening rate (% of patients screened), performed by a speech therapist using the Gugging Swallowing Screen (GUSS) (Trapl et al., 2007), in-hospital physiotherapy (% of patients), and length of hospital stay.

Because NIHSS was not routinely documented during the BSU period, two independent researchers estimated scores from neurological descriptions, and interrater agreement was assessed. The main outcome was obtained in the outpatient clinic for around 80% of patients; those not returning were evaluated by trained staff via telephone. When 90-day mRS was unavailable, mRS at hospital discharge was used as a substitute.

### Statistical analysis

Statistical analyses were performed using SPSS 28.0.1 (International Business Machines Corporation (IBM), Armonk, New York, United States). Descriptive statistics included proportions, means, standard deviations, medians, and interquartile ranges. Baseline characteristics and outcomes were compared using two-way ANOVA. Logistic regression was used to identify predictors of death while adjusting for confounders. Associations between discharge mRS distributions across groups were evaluated with Pearson's chi-squared test, and differences in 90-day mRS distributions were assessed using the Kruskal–Wallis test.

## Results

### Study sample and baseline characteristics

A total of 1,889 patients were included: BSU (*n* = 226), ASU (*n* = 1,073), and CSU (*n* = 590). Age, sex, and race were comparable across groups. Hemorrhagic stroke was proportionally more frequent in the BSU phase. Hypertension was less common in ASU, and smoking was more prevalent in ASU and CSU. Baseline NIHSS was lower in BSU compared with ASU and CSU (7.1 ± 6.1, 7.8 ± 7.5, and 8.1 ± 7.5; *p* < 0.001; Table 1). Missing baseline NIHSS occurred in 47 (20.8%) patients in BSU, 10 (0.9%) in ASU, and 7 (1.2%) in CSU. Interrater agreement for retrospectively estimated NIHSS scores during the BSU period was high [ICC_(2, 1)_ = 0.84], with 97.4% of paired ratings within ±2 points.

**Table 1:** Baseline characteristics.

Variable	Before stroke unit (*n* = 226)	Acute stroke unit (*n* = 1073)	Comprehensive stroke unit (*n* = 590)	*p*-value
*Demographic features*				
Age, mean ± SD	64.2 ± 14	64.1 ± 14	64.8 ± 14	0.91
Female Sex (%)	53.9	48.6	52.6	0.19
White race (%)	81.8	84.6	80.8	0.74
*Stroke type*				
Ischemic stroke (%)	68.8	82.2	82.5	< 0.001
Transient ischemic attack (%)	13.7	6.5	7.1	< 0.001
Hemorrhagic stroke (%)	16.8	8.4	9.2	< 0.001
Others (%)	2.7	2,9	1.2	0.08
*Risk factors*				
Hypertension (%)	88.5	80.2	87.3	< 0.001
Diabetes (%)	36.3	30.5	31.9	0.55
Current or previous smoking (%)	20.2	42.3	45.8	< 0.001
Ischemic cardiopathy (%)	14.6	19.1	18.1	0.60
Heart failure (%)	9.5	11.2	7.2	0.55
Atrial fibrillation (%)	20.5	15.7	20.1	0.06
Previous stroke (%)	30.0	31.9	19.4	0.09
*Characteristics at admission*				
Arrival to the hospital by ambulance (%)	17.8	40.3	52.0	< 0.001
Baseline NIHSS score (mean ± SD)	7.0 ± 6.1	7.8 ± 7.5	8.1 ± 7.5	< 0.001

### Clinical outcomes and predictors of recovery

**Figure 3:**
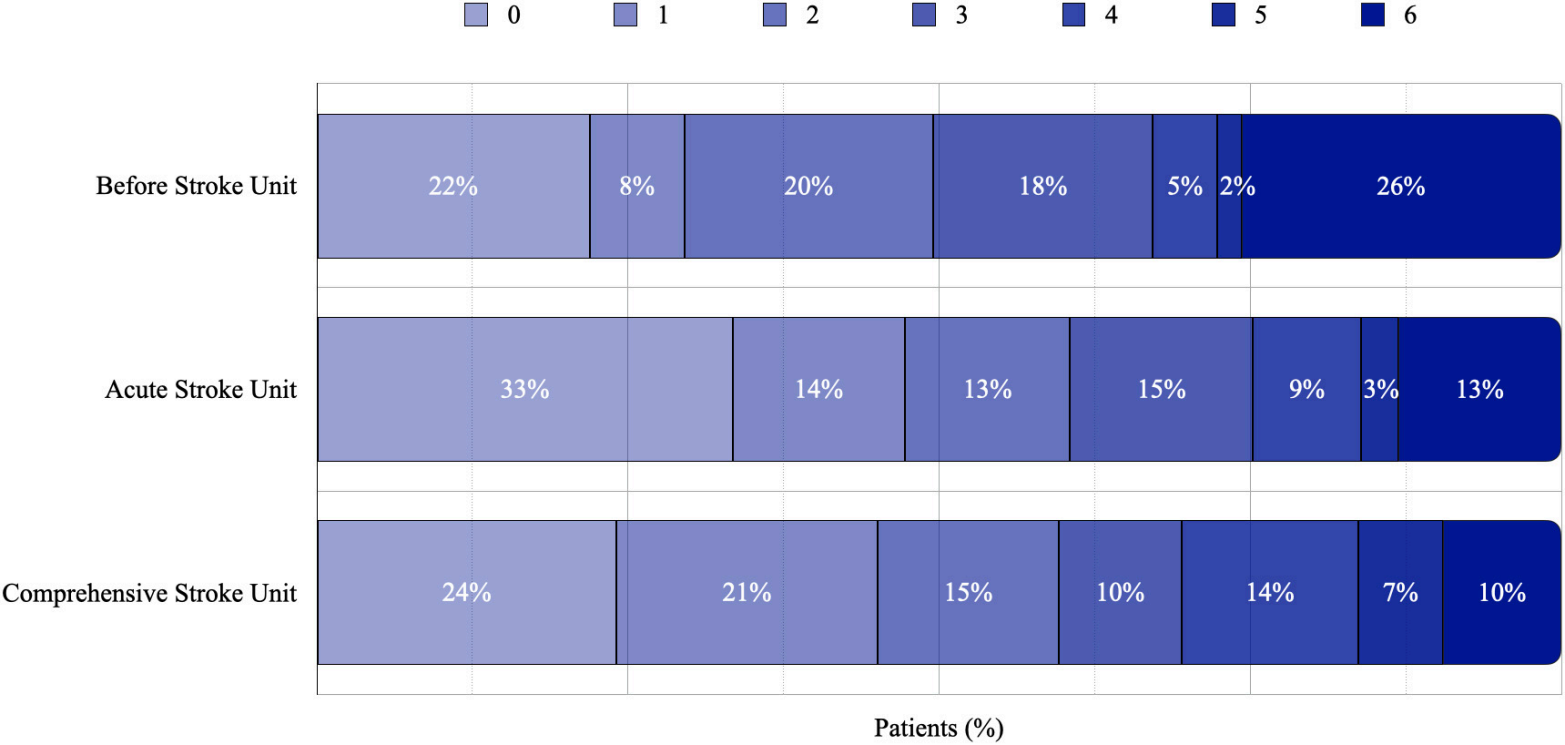
Ordinal shift analysis of the modified Rankin scale (mRS) score at 90 days after stroke in each model of care.

Main clinical outcomes are presented in Table 2. The proportion of patients achieving mRS 0–1 at 90 days was 27.0% (BSU), 47.1% (ASU), and 42.5% (CSU; *p* < 0.001). The 90-day mRS was missing in 16 (7.1%) in BSU, 4 (0.4%) in ASU, and 33 (5.6%) in CSU patients. Ordinal shift analysis of the mRS score at 90 days after stroke in each model of care is depicted in Figure 3. Using mRS at discharge as the main outcome, rates of mRS 0–1 were 11.9% (BSU), 40.3% (ASU), and 38.6% (CSU; *p* < 0.001), while mRS 0–2 rates were 19.5%, 53.4%, and 48.3%, respectively (*p* < 0.001). Functional independence (mRS 0–2) increased from 45.6% in BSU to 60.3% (ASU) and 56.3% (CSU; *p* < 0.001). Ninety-day case fatality decreased across periods (24.3%, 10.2%, and 7.7%; *p* < 0.001), and remained higher in BSU when ischemic and hemorrhagic strokes were analyzed separately.

**Table 2:** Main outcomes.

Variable	Before stroke unit (*n* = 226)	Acute stroke unit (*n* = 1,073)	Comprehensive stroke unit (*n* = 590)	*p*-value
*Efficacy and safety outcomes*				
Minimal or no disability at 90 days (mRS 0–1) (%)	11.9	40.3	38.6	< 0.001
Functional independence at 90 days (mRS 0–2) (%)	19.5	53.4	48.3	< 0.001
Mortality at 90 days (%)	24.3	10.2	7.7	< 0.001
Ischemic stroke (%)	15.2	9.2	7.9	0.02
Hemorrhagic stroke (%)	52.6	23.3	13.7	< 0.001

In logistic regression, predictors of death included age (OR 1.04/year; 95% CI 1.02–1.05; *p* < 0.001), baseline NIHSS (OR 1.19/point; 95% CI 1.16–1.23; *p* < 0.001), and hemorrhagic stroke (OR 1.68; 95% CI 1.30–2.17; *p* < 0.001). IV thrombolysis (OR 0.38; 95% CI 0.19–0.79; *p* = 0.009) and SU care (OR 0.28; 95% CI 0.17–0.47; *p* < 0.001) were independently associated with reduced case fatality.

In the model evaluating excellent functional outcome (mRS 0–1), SU care remained strongly associated with favorable recovery (OR 3.60; 95% CI 1.90–6.82; *p* < 0.001). Younger age and lower NIHSS were also independent predictors (OR 0.97/year; 95% CI 0.96–0.98; *p* < 0.001; and OR 0.75/point; 95% CI 0.72–0.78; *p* < 0.001). Ischemic stroke was associated with higher odds of excellent outcome compared with hemorrhagic stroke (OR 1.94; 95% CI 1.10–3.44; *p* = 0.023). Thrombolysis was the strongest predictor (OR 6.19; 95% CI 3.78–10.15; *p* < 0.001).

### Quality-of-care endpoints

Quality-of-care indicators (Table 3) improved progressively. Ambulance arrivals were significantly more common in ASU and CSU than BSU (40.3%, 52.0%, and 13.7%, respectively; *p* < 0.001). Door-to-CT time decreased from 527 ± 239 min (BSU) to 170 ± 196 (ASU) and 107 ± 165 (CSU; *p* < 0.001). Among patients arriving within the thrombolysis window, door-to-CT time was 15 ± 17 min in ASU and 12 ± 10 min in CSU (*p* = 0.07). Thrombolysis rates increased (0%, 14.1%, and 22.2%; *p* < 0.001). Swallowing and physiotherapy assessments became routinely available only after CSU implementation. Post-stroke pneumonia decreased from 29.4 to 16.3% and 12.5% (*p* < 0.001). Median length of stay fell from 13 [7–21] days in BSU to 8 [4–14] in ASU and 7 [4–13] in CSU (*p* = 0.034).

**Table 3:** Quality of stroke care indicators.

Variable	Before stroke unit (*n* = 226)	Acute stroke unit (*n* = 1,073)	Comprehensive stroke unit (*n* = 590)	*p*-value
*Quality of care*				
Door-to-CT time, min (mean ± SD)	527 ± 239	170 ± 196	107 ± 165	< 0.001
Alteplase administration among ischemic stroke patients (%)	0	14.1	22.2	< 0.001
In-hospital physiotherapy (% of patients)	0	3.5	90.5	< 0.001
In-hospital dysphagia screening (% of patients)	0	0	75	< 0.001
In-hospital pneumonia rate (%)	29.4	16.3	12.5	< 0.001
Length of hospital stay, days [median (IQR)]	13 [7–21]	8 [4–14]	7 [4–13]	0.034

## Discussion

### Study sample and baseline characteristics

Changes in baseline characteristics across study periods merit careful consideration. The observed variation in stroke subtype distribution likely reflects evolving regional stroke care pathways over time, particularly as the study center progressively assumed a more central role in acute stroke management. Most comorbidities were similarly distributed across groups, although hypertension was more prevalent in the ASU group, and differences in smoking status were noted—likely influenced by underreporting in earlier periods.

Importantly, there was a progressive increase in the proportion of patients arriving by ambulance, suggesting improvements in pre-hospital care organization, public awareness of stroke symptoms, and healthcare system responsiveness. These changes likely contributed to differences in case mix and baseline stroke severity across study periods and should be considered when interpreting temporal trends in outcomes.

### Clinical outcomes and predictors of recovery

Overall, our findings demonstrate a substantial improvement in functional outcomes and survival following the progressive implementation of structured stroke care. The proportion of patients achieving minimal or no disability at 90 days, as well as those maintaining functional independence despite residual deficits, increased significantly across care models.

Interestingly, the transition from ASU to CSU was not associated with further improvements in the proportion of excellent or good functional outcomes, despite the introduction of multidisciplinary care and early inpatient rehabilitation. This finding is likely explained, at least in part, by differences in case mix, as patients treated during the CSU period presented with higher baseline NIHSS scores. In addition, limited access to sustained post-discharge rehabilitation may have attenuated potential long-term functional gains. Notably, an additional analysis using discharge mRS confirmed the overall pattern of improved functional status across care models.

In contrast, case fatality rates declined consistently with each step in care organization, with the greatest reduction observed following CSU implementation. This effect remained significant in subgroup analyses by stroke subtype. In our investigation, SU care emerged as an independent predictor of reduced case-fatality after adjustment for major confounders, including age, baseline NIHSS, stroke subtype, and thrombolytic therapy. Notably, the magnitude of the association between SU care and reduced case-fatality was comparable to—and slightly greater than—that observed for thrombolysis, likely reflecting its broader population-level applicability. Overall, SU care was associated with a 62% relative reduction in 90-day case fatality, with rates decreasing from 24.3% outside SU to 9.3% within SU care.

The marked reduction in case-fatality, particularly among patients with hemorrhagic stroke, likely reflects improvements in the overall organization and intensity of acute care rather than the effect of any single intervention. In line with this, our study demonstrated a reduction in hemorrhagic stroke case fatality from 53% in the BSU period to 23% after ASU implementation and further to 14% following CSU establishment.

This interpretation is supported by prior studies demonstrating that structured SU care and care bundles improve outcomes in intracerebral hemorrhage. Admission to SU is associated with lower mortality and better functional outcomes compared with non-specialized care (Ungerer et al., 2020). Similarly, standardized care bundles have been linked to significant reductions in case fatality (Parry-Jones et al., 2019), and higher intensity of acute care is independently associated with lower short-term mortality (Martinez et al., 2020). Together, these findings support the concept that the organization and quality of early stroke care are key determinants of clinical outcomes.

Our results are consistent with findings from other LMIC settings, where SU implementation has been associated with improved outcomes, including reduced mortality and improved functional independence in Guinea (Cisse et al., 2019), the Philippines (Navarrro et al., 2021), South Africa (de Villiers et al., 2009), Argentina (Sposato et al., 2008), and Israel (Koton et al., 2005). Moreover, randomized evidence summarized in a recent meta-analysis (Langhorne and Ramachandra, 2020) supports the effectiveness of SU care across diverse healthcare systems.

Supporting evidence from Brazil further reinforces the importance of organized stroke care at a systems level. A population-based study demonstrated significant regional variation in stroke incidence, case fatality, and functional outcomes, with worse results observed in areas with lower socioeconomic development and limited access to structured stroke services (Dos Santos et al., 2022). Additional epidemiological data indicate that, although proportional stroke mortality has declined over time, the absolute burden of stroke continues to rise in Brazil, reflecting demographic changes and persistent healthcare disparities (Mpuhua et al., 2025). Furthermore, clinical studies have shown that stroke severity and early physiological parameters are key determinants of outcomes, underscoring the importance of structured, protocol-driven care (Kuster et al., 2014).

### Quality-of-care endpoints

The progressive implementation of SU care was associated with measurable improvements in multiple quality-of-care indicators. The introduction of an ASU, supported by standardized protocols for rapid evaluation and physiological optimization—including blood pressure, glucose, temperature, and oxygen control—was associated with faster diagnostic workflows and increased thrombolysis rates. The subsequent transition to a CSU further expanded care delivery through multidisciplinary involvement, including systematic swallowing assessments and early physiotherapy.

This stepwise increase in care complexity was accompanied by reductions in disability, pneumonia incidence, and length of hospital stay compared with the baseline period. These findings are consistent with reports from other LMICs, where SU implementation has been associated with reductions in complications, infections, and hospital length of stay, including studies from India (John et al., 2021), Guinea (Cisse et al., 2019), South Africa (de Villiers et al., 2009), Argentina (Sposato et al., 2008), and Israel (Koton et al., 2005).

Advances in stroke care over recent decades have been driven both by improvements in general supportive care and by the development of reperfusion therapies. While thrombolysis and thrombectomy have transformed outcomes in selected patients with ischemic stroke, hemorrhagic stroke remains without widely effective targeted therapies. Nevertheless, improvements in structured supportive care have translated into better outcomes in this subgroup. Evidence from the INTERACT2 study (Anderson et al., 2013) and the INTERACT3 trial (Ma et al., 2023) highlights the impact of protocolized, bundled care in improving outcomes in intracerebral hemorrhage, reinforcing the relevance of organized SU care.

### Health system implications and broader impact

Beyond acute management, SU contributes to improvements across the entire stroke care continuum. Concentration of expertise facilitates more comprehensive etiological investigation, implementation of secondary prevention strategies, and structured discharge planning. In addition, SU plays an important role in professional training, caregiver engagement, and community education, promoting earlier recognition of stroke symptoms and timely access to care. However, limited access to outpatient rehabilitation remains a major challenge in LMICs and may constrain long-term functional recovery (Langhorne et al., 2018).

The implementation of SU in resource-limited settings is often challenged by financial constraints. However, higher costs in stroke care in LMICs are largely driven by stroke severity and prolonged hospitalization (Kaur et al., 2014). By improving acute management, reducing complications, and shortening hospital stays, SU may represent a cost-effective strategy even in constrained environments, as supported by data from high-income settings such as the South London Stroke Register (Saka et al., 2009).

In Brazil, stroke remains the leading cause of death (Brasil, 2024), with most patients treated within the public health system. The implementation of a national stroke policy in 2012 (da Saúde, 2012a,b; Martins et al., 2013, 2023) marked a major step forward by introducing thrombolysis and formalizing stroke center organization, including ASUs and CSUs, alongside improved funding mechanisms. More recently, the incorporation of mechanical thrombectomy into the public system (da Saúde, 2023) represents an additional advance in stroke care. Together, these initiatives, along with global programs such as the Angels Initiative (Caso et al., 2023), highlight the importance of coordinated efforts to expand access to high-quality stroke care.

Taken together, our findings indicate that the implementation of organized SU care is a highly effective strategy to improve outcomes at both the individual and population levels, particularly in settings with constrained resources and significant healthcare disparities.

### Strengths and limitations

This longitudinal observational study is based on real-world data from a resource-constrained environment, which enhances the external validity and applicability of its findings to similar settings in Brazil and other LMICs. The large sample size further strengthens the reliability of statistical estimates and supports the consistency of the observed associations.

Nevertheless, the retrospective and observational design inherently limits internal validity and introduces susceptibility to selection, confounding, and measurement biases. In addition, the three care models evaluated (BSU, ASU, and CSU) represent sequential phases of care implementation rather than exchangeable cohorts, limiting direct comparability between groups and precluding causal inference.

A key limitation is the nonsystematic registry of information, particularly during the BSU period, which restricted data availability. This was mitigated through independent estimation of baseline NIHSS, structured handling of missing data, and additional subanalyses. Interrater agreement for NIHSS estimation was good, supporting the reliability of reconstructed values. The use of discharge mRS for cases lacking 90-day follow-up may have biased results toward smaller differences between care models, thereby reinforcing the robustness of the observed effects.

Changes in regional stroke care pathways over time may also have introduced referral bias. As the study center progressively became a regional hub for acute stroke therapies, particularly reperfusion strategies, there may have been preferential referral of patients with suspected ischemic stroke, while some hemorrhagic cases—potentially more severe and associated with higher case fatality—may have been managed in other institutions. This shift could have influenced the observed distribution of stroke subtypes and clinical outcomes across study periods.

Furthermore, the evolution of prehospital care and triage systems likely influenced case mix and baseline stroke severity at admission. Improvements in emergency medical services, increased training of healthcare professionals, greater public awareness of stroke symptoms, and the widespread implementation of time-sensitive treatment protocols may have led to earlier hospital arrival and changes in patient selection over time. These factors may have contributed to differences in baseline characteristics and outcomes between care models.

The study population largely reflects the demographic profile of the Porto Alegre metropolitan area—predominantly individuals in their sixties and of European ancestry—supporting regional representativeness but limiting generalizability to more diverse populations. In addition, ambulance arrival rates may be overestimated due to interhospital transfers from neighboring municipalities.

A higher proportion of hemorrhagic strokes in the BSU period may also have contributed to poorer outcomes in this group. However, baseline NIHSS was slightly lower during this period, a difference of uncertain clinical relevance that may reflect the inclusion of transient ischemic attacks or differences in case ascertainment. Importantly, the observed reduction in case fatality remained significant in subgroup analyses of hemorrhagic stroke, supporting the consistency of the findings.

Although secular improvements in overall medical care over time cannot be fully excluded as contributing factors, they are unlikely to account for the magnitude and consistency of the effects observed across multiple outcomes and analyses.

## Conclusion

The progressive implementation of structured SU care was associated with substantial improvements in survival and functional outcomes in a real-world, resource-constrained setting. These benefits were consistent across both ischemic and hemorrhagic stroke subtypes and were primarily driven by enhancements in the organization, quality, and intensity of acute care rather than by isolated interventions.

Our findings reinforce SU care as a high-impact, scalable strategy with population-level benefits, particularly in LMICs where access to specialized care remains limited. Expanding the availability of CSU, alongside strengthening prehospital systems and access to rehabilitation, represents a critical step toward reducing stroke-related mortality, disability, and healthcare disparities.

## Data Availability

Datasets represent anonymized data from a public hospital, under responsibility of the investigators. They can be made available upon reasonable request. Requests to access these datasets should be directed to Sheila Cristina Ouriques Martins, sheila.ouriques.martins@gmail.com.
